# CRISPR/Cas9-Mediated α-ENaC Knockout in a Murine Pancreatic β-Cell Line

**DOI:** 10.3389/fgene.2021.664799

**Published:** 2021-04-01

**Authors:** Xue Zhang, Lihua Zhao, Runbing Jin, Min Li, Mei-Shuang Li, Rongfeng Li, Xiubin Liang

**Affiliations:** ^1^Department of Pathophysiology, Nanjing Medical University, Nanjing, China; ^2^Jiangsu Key Laboratory of Xenotransplantation, Nanjing Medical University, Nanjing, China; ^3^Department of Nephrology, The Affiliated Sir Run Run Hospital of Nanjing Medical University, Nanjing, China

**Keywords:** CRISPR/Cas9, pancreatic β-cells, gene knockout, MIN6 cells, α-ENaC

## Abstract

Many ion channels participate in controlling insulin synthesis and secretion of pancreatic β-cells. Epithelial sodium channel (ENaC) expressed in human pancreatic tissue, but the biological role of ENaC in pancreatic β-cells is still unclear. Here, we applied the CRISPR/Cas9 gene editing technique to knockout α-ENaC gene in a murine pancreatic β-cell line (MIN6 cell). Four single-guide RNA (sgRNA) sites were designed for the exons of α-ENaC. The sgRNA1 and sgRNA3 with the higher activity were constructed and co-transfected into MIN6 cells. Through processing a series of experiment flow included drug screening, cloning, and sequencing, the α-ENaC gene-knockout (α-ENaC^−/−^) in MIN6 cells were obtained. Compared with the wild-type MIN6 cells, the cell viability and insulin content were significantly increased in α-ENaC^−/−^ MIN6 cells. Therefore, α-ENaC^−/−^ MIN6 cells generated by CRISPR/Cas9 technology added an effective tool to study the biological function of α-ENaC in pancreatic β-cells.

## Introduction

Diabetes mellitus (DM) is usually characterized by an insulin deficiency due to pancreatic β-cell dysfunction including insulin secretion and insulin action, and disorder of insulin secretion promotes diabetes progression (Roden, 2016; Chatterjee et al., 2017). DM frequently coincides with many clinical features of hypertension. Hypertension is well-known as a high-risk factor for both microvascular and macrovascular chronic diabetic complications (Yamazaki et al., 2018). More research results show that clinically refractory hypertension (resistant hypertension), including salt-sensitive hypertension and obesity-related hypertension, are all closely related to the overactivation of epithelial sodium channel (ENaC) expression and function (Bubien, 2010; Pitzer et al., 2020). ENaC is a continuously activated non-voltage-dependent sodium ion channel that can be specifically inhibited by amiloride, so it is called amiloride-sensitive sodium ion channel (Kellenberger and Schild, 2002). Studies have shown that ENaC includes four subunits α, β, γ, and δ, which are used by SCNN1A, SCNN1B, SCNN1G, and SCNN1D gene codes (Butterworth et al., 2009). The α subunit of ENaC (α-ENaC) plays a major role in sodium ion transport, while the β and γ subunits (β-ENaC and γ-ENaC) alone do not support sodium transport and only play a regulatory role. ENaC is widely expressed in various organs and tissues throughout the body. In the kidney, ENaC is mainly distributed in the distal convoluted tubules and collecting ducts of the distal nephrons, and acts as a rate-limiting effect on sodium reabsorption by mediating the transmembrane transport of sodium ions. This plays an important role in maintaining sodium’s own balance, extracellular fluid volume and stabilizing blood pressure (Rossier, 2014; Hanukoglu and Hanukoglu, 2016). Through the protein expression database,^1^ we found that α-ENaC is clearly expressed in human pancreatic tissue, and its expression score in the pancreas is the same as that in other tissues such as kidney. However, the mechanism of α-ENaC in pancreatic β-cell is not clear. Thereby, the cell tool for studying the function of α-ENaC in β-cell biology is essential.

The CRISPR/Cas9 mediated gene editing technology is one of the most important methods in gene function research of reverse genetics (Wang et al., 2016). The specific DNA editing of targeted genes can be realized *via* DNA double-strand break (DSB) and non-homologous end joining-mediated DNA repair (NHEJ) after the single-guide RNA (sgRNA)-directed Cas nucleases cutting of target DNA (Gori et al., 2015; Fang et al., 2018). Among the current gene editing tools, including zinc finger nuclease (ZFN) and transcriptional activator like effector nuclease (TALENs), the CRISPR/Cas9 system is the most efficient regarding the gene knockout and the easiest to construct and use (Ni et al., 2014). In this study, we design to delete the α-ENaC gene from a mouse pancreatic β-cell line genome (MIN6 cell) *via* the CRISPR/Cas9 technique, and the morphology and viability of MIN6 cells after α-ENaC knockout are evaluated *in vitro*. The derived α-ENaC deficient MIN6 cell line will become an effective tool for elucidating the α-ENaC function and its molecular mechanism in diabetes mellitus.

## Materials and Methods

### Design of sgRNA and Synthesis of Oligonucleotide Chains

α-ENaC gene sequence was searched on GenBank and α-ENaC sequence was analyzed on the coding region. On the basis of the first exon sequence, the sgRNA sequences were designed with the online tool,^2^ and a BpiI restriction site was added in the each sgRNA sequence. The complementary phosphorylated DNA oligos of the sgRNAs were synthesized, and annealed to form the double-strand DNA using thermal conditions: at 37°C for 30 min, and at 95°C for 5 min, followed by decreasing at 5°C/min to 25°C.

### Construction of Cas9 Expression Vector

The PX330 vector (#42230, Addgene, United States), Cas9 expression plasmid with pX330-U6-Chimeric-BB-CBh-hSp-Cas9 construct, was linearized by BpiI at 37°C for 30 min. The product with sticky ends was purified by agarose gel electrophoresis and gel extraction. The respective phosphorylated double-strand DNA oligos of the sgRNA was ligated to the linearized PX330 vector by T4 DNA ligase (TAKARA), and then the ligation product (α-ENaC-Cas9/sgRNA) was transferred to DH5α (TianGen, China) receptive cells for transformation, and coated on the LB medium plate for ampicillin resistance as described previously (Wang et al., 2019). Single colonies were selected and shaken for culture, and small plasmids were extracted and sequenced for identification.

### Cell Culture and Transfection

The reagents for cell culture were all purchased from Gibco (Oakland, United States). MIN6 cells (RRID: CVCL_0431) were cultured in DMEM with 4.5 g/L glucose and 15% FBS and 50 μmol/L beta-mercaptoethanol in a 37°C incubator with 5% CO_2_. One day before cell transfection, the MIN6 cells were thawed and seeded into 6-well plates and cultured up to the 90% confluence. 1 × 10^6^ MIN6 cells were transfected with 5 μg α-ENaC-Cas9/sgRNA plasmids *via* the P3 Primary Cell 4D-Nucleofector with X Kit (Amaxa Biosystems/Lonza, United States) according to the manufacturer’s instructions, and the transfected cells were cultured in the MIN6 culture medium.

### T7E1 Cleavage Assay and Sequence Analysis

MIN6 cells transfected with or without α-ENaC-Cas9/sgRNA plasmids were cultured for 48 h. The DNA extraction kit (TianGen, China) was used to extract the genomic DNA of the transfected MIN6 cells, and the PCR primers (forward: 5'-TCG CTG TGA CCA CTT CGC TCT G-3'; reverse: 5'-CAC AGT GAC GGC AGG GAA GAC CAG-3') were synthesized to amplify the genomic region spanning the CRISPR target sites. The PCR product size should be 655 bp. The PCR conditions were 98°C for 30 s, followed by 30 cycles of 98°C for 5 s, 66°C for 10 s, and 72°C for 20 s; and then 72°C for 2 min and held at 4°C. An EnGen® Mutation Detection kit (NEB, United States) was used to perform the T7E1 cleavage assay according to the manufacturer’s protocol as described previously (Fang et al., 2018). Briefly, a total of 200 ng of the purified PCR product were denatured and annealed in NEB buffer 2 using the following conditions: 95°C for 5 min, 95–85°C ramping at −2°C/s, 85–25°C ramping at −0.1°C/s and holding at 4°C. After reannealing, the heteroduplex PCR products were digested with 1 μl of T7 endonuclease I for 15 min at 37°C and then run on an agarose gel stained with ethidium bromide. Meanwhile, the remaining purified PCR products were subjected to Sanger sequence analysis. The sequencing results were compared with wild-type (WT) MIN6 cells *via* the online tool.^3^

### Acquisition of Monoclonal Cells

The MIN6 cells were transfected with two more efficient α-ENaC-Cas9/sgRNA plasmids (sgRNA1 and sgRNA3) and 0.8 μg pCMV-TD-Tomato vector plasmids as mentioned above. The transfected cells were seeded in 10 cm dishes at a density of 1 × 10^4^ cells per well and recovered for 48 h in the MIN6 culture medium. After recovery, 800 μg/ml of G418 (Gibco, United States) was added into the culture medium and maintained for about 14 days to select the cell colonies with the neomycin resistant gene as described previously (Wang et al., 2019). Individual cell colonies were collected by trypsinization and cultured in 96/48-well plates and sub-cultured in 12-well plates. Twenty percentage of cells from each resistant cell clones was, respectively, collected to extract the genomic DNA for the identification experiment of α-ENaC-knockout, most of the rest cells (approximately 80%) were cryopreserved in cell freezing medium for future use.

### Identification of α-ENaC-Knockout in MIN6 Cells

Genomic DNA was extracted from previous resistant cell clones and amplified by PCR amplification using primers (as described in T7E1 assay). The PCR products were detected by agarose gel electrophoresis, and the remaining PCR products were subjected to Sanger sequence analysis. The sequencing results were compared with α-ENaC gene. The PCR products with sequence inconsistency were gel purified and cloned into pMD18-T vector (TAKARA), respectively. Twelve positive plasmid clones were randomly picked up and sequenced.

### Off-Target Analysis

While the sgRNA sequences were designed, the potential off-target sites (OTSs) of the two Cas9-α-ENaC-sgRNAs (sgRNA1 and sgRNA3) were predicted using the online tool.^4^ In α-ENaC^−/−^ MIN6 cell line, the top 10 OTSs for each sgRNA were selected in Supplementary Table S1, and then the genomic regions covering the OTSs were PCR amplified using the primers listed in Supplementary Table S2. These PCR products were subjected to Sanger sequence analysis.

### Western Blot Analysis

The equal amount of protein sample from WT or α-ENaC^−/−^ MIN6 cells were resolved by 10% SDS-PAGE, and then were transferred to polyvinylidene difluoride (PVDF) membranes. The PVDF membrane was blocked at room temperature for 1–2 h by 5% (w/v) skim milk in TBST. The blots were incubated with α-ENaC antibody (StressMarq Biosciences, Canada) at 1:1,000 dilution for 2 h at room temperature, followed by an anti-rabbit secondary antibody (1:8,000). The result was detected by enhanced chemiluminescence reagent. β-Actin was used as housekeeping gene.

### CCK8 Assay

Cell Counting Kit-8 (CCK8; APExBIO, United States) was used to measure cell viability. WT or α-ENaC^−/−^ MIN6 cells were seeded in 96-well plates at 1 × 10^4^ cells per well (five replicate wells). After culturing for 24, 48, and 72 h, 10 μl of CCK-8 solution was added to each well and incubated for 2 h, cellular viability was determined by measuring the absorbance at 450 nm.

### Insulin Content Assay

Wild-type or α-ENaC^−/−^ MIN6 cells were seeded in a 24-well plate, after 1 h incubation in glucose-free Krebs-Ringer bicarbonate (KRB) buffer (115 mmol/L NaCl, 4.7 mmol/L KCl, 1.2 mmol/L MgSO_4_·7H_2_O, 1.2 mmol/L KH_2_PO_4_, 20 mmol/L NaHCO_3_, 16 mmol/L HEPES, 2.56 mmol/L CaCl_2_, and 0.2% BSA), the cells were cultured for 1 h in KRB contained stimulatory glucose (16 mmol/L) and rinsed with PBS. Each well was added with 500 μl acid ethanol extract (1.4% hydrochloric acid in 74% anhydrous ethanol) and incubated at 4°C overnight. The supernatants were obtained for insulin concentration determination using a commercial Insulin ELISA kit (Crystal Chem Inc., United States) according to the manufacturer’s protocol. These intracellular insulin content results were normalized to the total protein concentration.

### Immunofluorescence Staining

Wild-type or α-ENaC^−/−^ MIN6 cells were grown in glass-bottom culture dishes at 37°C with 5% CO_2_, after 24 h, cells were fixed (4% paraformaldehyde), permeabilized (5% Triton X-100), blocked (1% bovine serum albumin), and then the cells were incubated with primary insulin antibody (Abcam) at 4°C overnight. Next, the fluorescent mouse IgG (Thermo Fisher) were incubated for 1 h at room temperature and nuclei were stained with 4',6-diamidino-2-phenylindole (DAPI; Abcam). Finally, the cells were observed under the fluorescence microscopy.

### Statistical Analysis

All data were presented as mean ± SEM and compared by Student’s *t*-test. Statistical analysis of the data was performed using statistical analysis software GraphPad Prism 6.0 (GraphPad Software, Inc. La Jolla, CA, United States). *p* < 0.05 was considered statistically significant.

## Result

### Construction of Cas9-sgRNA Vectors and Screening of the Targeting Efficiency

**Figure 1:**
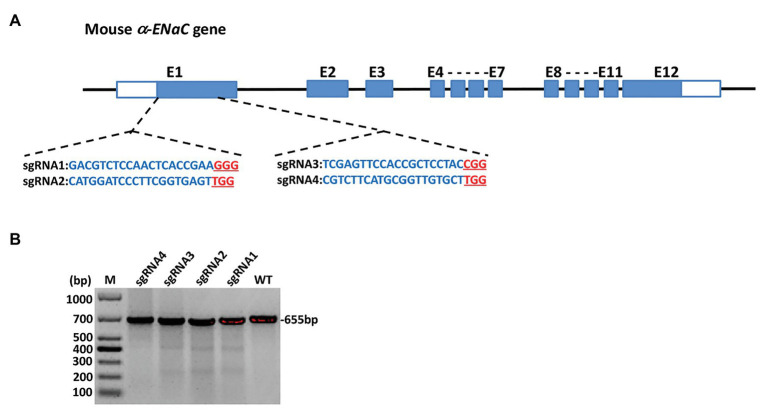
Generation and validation of α subunit of epithelial sodium channel (α-ENaC) knockout MIN6 cells. **(A)** The elements of mouse α-ENaC gene and the location and sequences of designed single-guide RNAs (sgRNAs). The α-ENaC-sgRNA1 and α-ENaC-sgRNA2 were located in the forepart of the first exon (coding sequence), and the α-ENaC-sgRNA3 and α-ENaC-sgRNA4 were located in the last segment of the first exon. **(B)** T7E1 assay for CRISPR/Cas9-mediated cleavage at target site in MIN6 cells. WT, PCR products of the wild-type MIN6 treated with T7E1; sgRNA1~4, PCR products of MIN6 transfected with Cas9/sgRNA 1~4 treated with T7E1; M, DL1000 DNA Marker.

The four sgRNAs (α-ENaC-sgRNA1, α-ENaC-sgRNA2, α-ENaC-sgRNA3, and α-ENaC-sgRNA4) were designed (Figure 1A) and double-stranded formed by program annealing. pX330-U6-Chimeric-BB-CBh-hSp-Cas9 plasmid was cut by BpiI enzyme, the annealed sgRNAs were ligated with the lined plasmid by T4 ligase, and the ligation products (Cas9-α-ENaC-sgRNA1, Cas9-α-ENaC-sgRNA2, Cas9-α-ENaC-sgRNA3, and Cas9-α-ENaC-sgRNA4) were sent to company for sequencing. The results showed that the four sgRNAs were inserted into pX330-Cas9 vector correctly, and the position, direction, and sequence of the inserted sequence were consistent with the expectation, which proved that the construction of Cas9-sgRNA expression vectors was completely correct. We further compared the targeting efficiency of the four sgRNA with T7E1 cleavage assay and sequence analysis after, respectively, transfecting them into the MIN6 cells. The T7E1 enzyme digestion results showed that Cas9-sgRNA1 and Cas9-sgRNA3 targeting on the α-ENaC gene were highly efficient (Figure 1B), and the sequence analysis showed their mutation efficiencies were 18.7 and 11.2%, respectively (Supplementary Figure S1). These results suggested that the targeting vectors Cas9-α-ENaC-sgRNA1 and Cas9-α-ENaC-sgRNA3 had higher targeting efficiency for knocking-out the first exon of mouse α-ENaC gene.

### Identification of MIN6 Cell Line With Stable α-ENaC Gene Knockout

**Figure 2:**
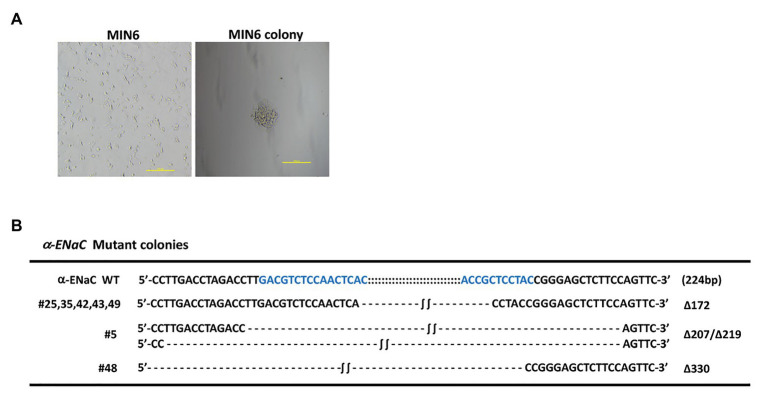
Identification of MIN6 cell line with stable α-ENaC gene knockout. **(A)** The cytomorphology of monoclonal positive MIN6 cell colony after G418 selection. **(B)** Genotypes of seven cell lines with large fragment biallelic deletion. α-ENaC WT, the wild-type α-ENaC sequence (224 bp) including the targeting regions of α-ENaC-sgRNA1 and α-ENaC-sgRNA3; ∆, deletion.

pX330-U6-Cas9-α-ENaC sgRNA1, pX330-U6-Cas9-α-ENaC sgRNA3, and PCMV-TD-tomato donor plasmid were co-transfected into MIN6 cells using nucleofection. After screening with G418, 32 monoclonal positive MIN6 cell colonies were obtained (Figure 2A). The genomes of the 34 positive cell lines and the wild-type cells were extracted, which were used as templates for PCR amplification of the DNA fragment of the first exon region of α-ENaC gene. The results indicated that there are seven cell lines with large fragment biallelic deletion, 12 wild-type cell lines, and the remaining cell lines were of other type (Supplementary Figure S2). The results of the further sequencing assays showed that the deleted large fragments in the seven cell lines were all from the first exon region of α-ENaC gene (Figure 2B). We found that there was a 172-base-deletion (Δ172) in five cell lines including #25, #35, #42, #43, and #49 compared with the original sequence of α-ENaC, which could change the open reading frame encoded by α-ENaC gene and thus terminate the translation of α-ENaC protein. However, #5 (Δ207/Δ219) and #48 (Δ330) cell lines might result in non-shift mutation since they had three times of base-deletion (Figure 2B).

**Figure 3:**
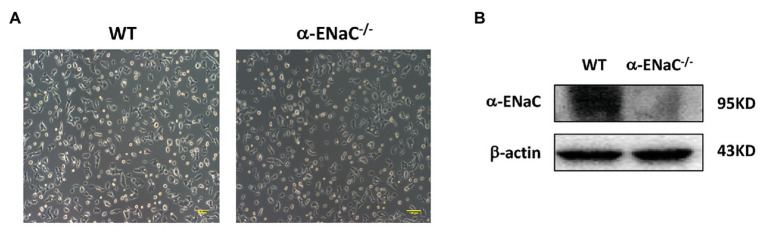
Verification of α-ENaC gene knockout in MIN6 cells. **(A)** The morphology in cultured WT and α-ENaC^−/−^ MIN6 cells (#42 cell line). **(B)** The expression of α-ENaC in α-ENaC^−/−^ MIN6 cells (#42 cell line) was detected by western blot.

All of five cell lines (#25, #35, #42, #43, and #49) grown on tissue culture plates spread and appeared of regular shape compared with the morphology of WT MIN6 cells. Furtherly, we found that #42 cell line grew at a rate comparable with WT MIN6 cells (data not shown) and had no signs of altered morphology or differentiation even after 20 passages (Figure 3A). Therefore, we chose #42 cell line to do further study. As shown in Figure 3B, α-ENaC expressed in WT MIN6 cells, but no signals of α-ENaC were detected in #42 cell line. The results indicated that the MIN6 cells with stable α-ENaC gene knockout were successfully constructed. The off-target analysis of #42 cell line was carried out in the top 10 OTSs for each sgRNA (Supplementary Tables S1,S2), and the results indicated that no off-target occurred at these 20 sites of #42 cell line.

### α-ENaC Deficiency Increased Cell Viability and Insulin Content in MIN6 Cells

**Figure 4:**
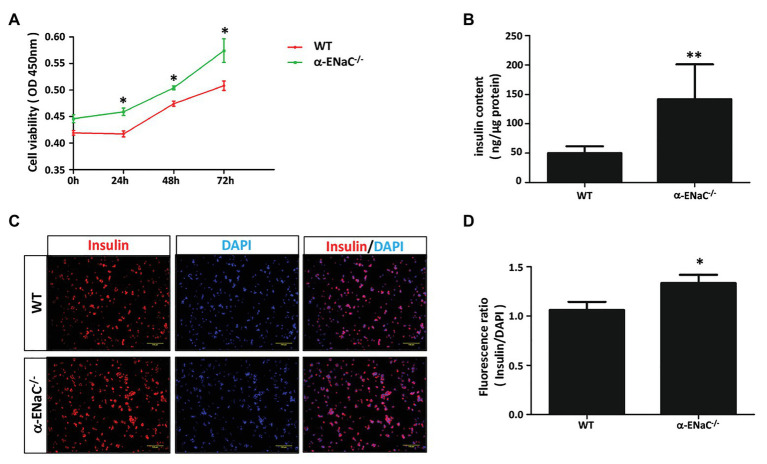
α-ENaC deficiency promotes cell viability and insulin content in MIN6 cells. **(A)** Knockdown of the α-ENaC gene enhanced the proliferation of MIN6 cells. **(B)** Knockdown of the α-ENaC gene enhanced the insulin content. **(C)** Immunofluorescence analyses were performed with an antibody against insulin. Representative images from five independent experiments are shown. **(D)** Quantification of insulin expression from experiments as shown in **(C)**. Data are expressed as mean ± SEM from five independent experiments; ^*^p < 0.05, ^**^p < 0.01.

To determine whether α-ENaC gene knockout affect the function of MIN6 cells, we examine cell viability and insulin content of #42 cell line. CCK8 assay showed that knockout of α-ENaC could dramatically promote MIN6 cells viability (Figure 4A). As shown in Figure 4B, compared with the control group, the insulin content was significantly increased in the α-ENaC^−/−^ MIN6 cells. Immunofluorescence staining was used with a specific antibody against insulin in WT or α-ENaC^−/−^ MIN6 cells. Insulin expression was significantly increased in α-ENaC^−/−^ cells (Figure 4C), which is consistent with the results described above. The data are summarized in Figure 4D.

## Discussion

In this study, α-ENaC gene in a mouse pancreatic β-cell line (MIN6 cell) was successfully knocked out using the CRISPR/Cas9 gene editing system. We successfully generated a tool of α-ENaC^−/−^ MIN6 cells to further study the function and mechanism of α-ENaC in pancreatic β-cells.

DNA sequencing results showed that α-ENaC gene occurred 172 bp deletion, and the mutation site was exactly the recognition sequence of sgRNA1and sgRNA3, which indicated that sgRNA1 and sgRNA3 accurately targeted exon of the α-ENaC gene, followed by Cas9 Nuclease produces DNA DSBs at the target site (Gasiunas et al., 2012). During the repair process of DNA in MIN6 cells, a deletion mutation occurs (Gratz et al., 2013). This mutation changes the open reading frame encoded by α-ENaC gene and terminates the protein translation. Before the emergence of CRISPR/Cas9 technology, RNA interference technology (RNAi) was commonly used to study gene function at the cellular level. RNAi, as a powerful tool for post-transcriptional silencing of gene expression, has made an important contribution to the analysis of gene functions and mechanisms, but with the development of technology, it has shown shortcomings of transientness and incompleteness (Carthew and Sontheimer, 2009).

The CRISPR/Cas9 system uses sgRNA to guide Cas9 nuclease to perform DNA-specific shearing at the target site to achieve the purpose of knocking out genes (Zhang et al., 2014). This study uses the CRISPR/Cas9 system to cause a deletion of α-ENaC gene in MIN6 cell genome level, thereby interfering with the expression of the α-ENaC gene, eliminating the shortcomings of transientness and incompleteness in the previous application of RNAi. Therefore, compared to RNAi, the CRISPR/Cas9 system is an effective tool more suitable for gene function research (Morgens et al., 2016; Smith et al., 2017).

ENaC is basically expressed at the collecting ducts of kidney to maintain body salt and water homeostasis. According to ENaC gene mutations that can cause severe dysfunction of these tissues in multi-system PHA (Enuka et al., 2012; Hanukoglu and Hanukoglu, 2016) and the function of ENaC expressed at a higher level in organs such as kidney, lung, respiratory tract, and sweat gland, the functional significance of ENaC is determined (Hanukoglu et al., 2017; Moore and Tarran, 2018). Although a search through the protein expression database^5^ found that α-ENaC is clearly expressed in human pancreatic tissue, there are not many research reports on the role of ENaC in the pancreas. In this experiment, α-ENaC gene-knockout in MIN6 cells (α-ENaC^−/−^) was generated and we obtained seven cell lines. We examined the cell viability and insulin content in α-ENaC^−/−^ MIN6 cells. Compared with WT MIN6 cells, the cell viability and insulin content were significantly increased in α-ENaC^−/−^ MIN6 cells. The other functions of α-ENaC^−/−^ MIN6 cells (e.g., glucose response and insulin secretion) will be performed in the future investigation. Therefore, our work added α-ENaC^−/−^ MIN6 cells to toolbox of pancreatic β-cells for α-ENaC biological field.

## Data Availability Statement

The original contributions presented in the study are included in the article/Supplementary Material, further inquiries can be directed to the corresponding authors.

## Author Contributions

RL and XL designed the experiments. XZ and LZ conducted the study. XZ, LZ, RJ, ML, and M-SL participated in the data collection and analysis. XZ, LZ, RL, and XL wrote the manuscript. All authors contributed to the article and approved the submitted version.

### Conflict of Interest

The authors declare that the research was conducted in the absence of any commercial or financial relationships that could be construed as a potential conflict of interest.

The reviewer XL declared a past co-authorship with one of the authors, RL to the handling editor.

## Footnotes

^1^https://www.proteinatlas.org

^2^https://zlab.bio/guide-design-resources

^3^https://tider.deskgen.com/

^4^https://zlab.bio/guide-design-resources

^5^https://www.proteinatlas.org
