# Supplementary material for: CRISPR/Cas9-Mediated α-ENaC Knockout in a Murine Pancreatic β-Cell Line
Source: Front Genet. 2021 Apr 1;12:664799. doi: 10.3389/fgene.2021.664799 (PMC8047203; doi:10.3389/fgene.2021.664799)
Supplement: Supplementary file 1 [file Data_Sheet_1.PDF]

Supplementary Data

Title

CRISPR/Cas9-mediated α-ENaC knockout in a murine pancreatic β cell line

Authors

Xue Zhang[1#], Lihua Zhao[2#], Runbing Jin[1], Min Li[1], Mei-Shuang Li[2], Rongfeng Li[2*] and Xiubin Liang[1, 3*]

Supplementary Figures

Supplementary Figure 1. The targeting efficiency of Cas9-sgRNA vectors detection via sequence analysis. The targeting efficiency of the four vectors (Cas9-α-ENaC-sgRNA1, Cas9-α-ENaC-sgRNA2, Cas9-α-ENaC-sgRNA3 and Cas9-α-ENaC-sgRNA4) to knockout the first exon of mouse α-ENaC gene were 18.7%, 5.7%, 11.2% and 7.1%, respectively.

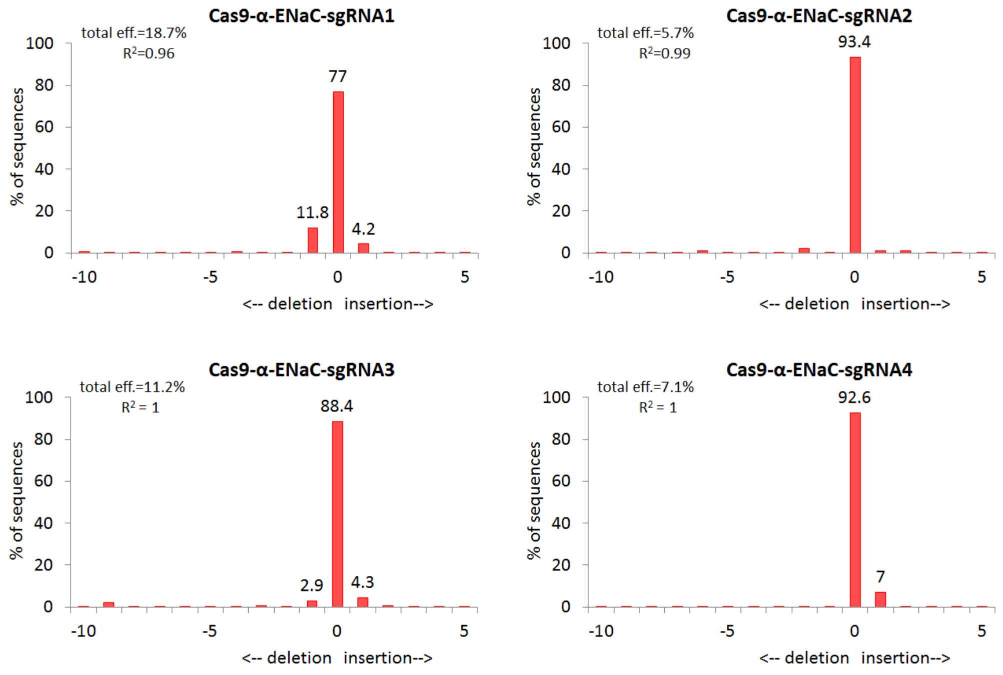

Supplementary Figure 2. The partial Agarose gel electrophoresis of PCR products with genomic DNAs of cell clones after G418 selection. M: DL1000 DNA marker; WT: PCR products with genomic DNAs of the wild-type MIN6 cell; # number: PCR products with genomic DNAs of cell lines after G418 selection.

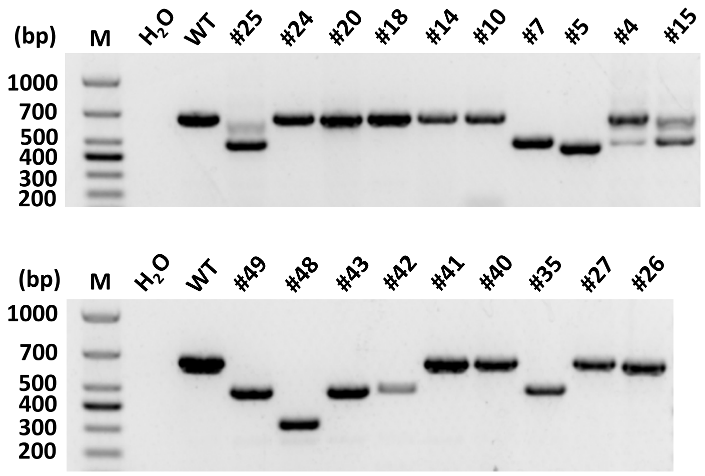

Supplemental Table 1. The potential off-target sequences

Off-target sites		Coordinates	Targets	PAM
sgRNA1	OTS1	chr17:93477396-93477415	GAAGTCTCTAACTCAACTAA	TGG
	OTS2	chr9:7003200-7003219	GAAGTTTCCAACTTACCAAA	AGG
	OTS3	chr15:35877104-35877126	TAAATCTCCAACTCACTGAA	GGG
	OTS4	chr9:40037670-40037692	AAAGTCTCAAACTCACCCAA	GGG
	OTS5	chr11:119906607-119906629	CATGTCTCAAAATCACCGAA	GGG
	OTS6	chr3:133876753-133876775	GGCGTTTCCAACCCACAGAA	GGG
	OTS7	chr1:39450921-39450943	CAAGTCTCCAACTCACCCAG	TGG
	OTS8	chr5:13563102-13563124	CACGTGTCCTACTCACCTAA	GGG
	OTS9	chr2:127476569-127476591	GAGATCTCCAAATCACAGAA	TGG
	OTS10	chr2:160157067-160157089	GAAGTCTCCAACTCACACAG	GGG
sgRNA3	OTS1	chr11:56951238-56951260	ACGATTTTCACCTCTCCTAC	AGG
	OTS2	chr2:32845887-32845909	TAAATTTCCACCTCTCCTAC	AGG
	OTS3	chr17:12209289-12209311	TGGAATTCTACTGCTCCTAC	GGG
	OTS4	chr9:70942045-70942067	CTGAGTCCCACCCCTCCTAC	TGG
	OTS5	chr7:47058412-47058434	CAGAGTTCCAGCCCTCCTAC	TGG
	OTS6	chr6:134856229-134856251	TCCACTTCCTCCGCTCCCAC	AGG
	OTS7	chr1:134591302-134591324	TCTATTTCCACTGCTCTTAC	TGG
	OTS8	chr3:27430677-27430699	TCTGGTTCCACAACTCCTAC	TGG
	OTS9	chr16:32557446-32557468	TCAATTTCCACCTCTCTTAC	TGG
	OTS10	chr11:69406594-69406616	TCGAGTGCCGCTGCTCCTAA	GGG

Supplemental Table 2. Primers for amplifying off-target sites.

Primer for OTSs to sgRNAs		Sequence (5′ -3′)	Amplicon (bp)
sgRNA1	OST1-for	TCCTTCTTTCCCTTCATTGGTTCC	785
	OST1-rev	TGACCACGAGAAAAGCACTGTTGA	
	OST2-for	TCAGGGCTTTGTTGTTGTTTGTTT	495
	OST2-rev	GCTAGACTCATGGGCTCATTGTTT	
	OST3-for	AAAAGAAGTCTGGGTCTGAGTGAA	671
	OST3-rev	ATCGCATTACAGATGGTTGTGAGT	
	OST4-for	AATGCGAAGAAGAAAGTTCACAGC	830
	OST4-rev	GATGAAGCCTACTTGATCGTGGTG	
	OST5-for	AGGGTATTGGTTTCTGGAACTGGA	697
	OST5-rev	GCAAAGCTGAAAGCCACGGAAGAG	
	OST6-for	GTGTTGATCCTAATTGGAGCAGAC	544
	OST6-rev	GAAAGTGGATAGACAAGGGAGATT	
	OST7-for	AACTGCCTTCTAACCCAATTCTAT	451
	OST7-rev	CTTTGGATGAGTCGGCTCCTATTT	
	OST8-for	TCCAGGCAAACCCAACTGACAGAC	746
	OST8-rev	CTTAGAGCACTATTAGCAGGAGCA	
	OST9-for	CTGTAGCTTTGCCCTTCTGTTGTC	624
	OST9-rev	GGACTGGGCTGGTGCTATCATTAA	
	OST10-for	TAGCTCACTGAGTAGGGCTGTAAC	719
	OST10- rev	TTAAGCAGGAAAGCACTTCAGGAT	
sgRNA3	OST1-for	TTGGAGGGAGTAGCAGTGAACAGA	762
	OST1-rev	CTGGGAAGAGTTGAACATGGAAAT	
	OST2-for	CAATTCAAGTAGCCTTTACAGCAC	657
	OST2-rev	CTGGTCCGAACCCTTAGAAATCCC	
	OST3-for	GAAGGGAGTGGGTGATGTGAAATG	638
	OST3-rev	CTTGGGAATCTAATGAGTCAGGGA	
	OST4-for	TTTTCAAAGGTGCCCAGGTAAGAT	586
	OST4-rev	GCTGTAGTCAGAGGTGGAAAGGGA	
	OST5-for	CCAGGGTTTGACGAGTTGAAGCAG	647
	OST5-rev	CAAGTACATGATGGCACAGCAGGA	
	OST6-for	ATAAAGGCTGAGAACCACGGGAGA	699
	OST6-rev	ATCGCAAACATGGTGGAAGGTAAA	
	OST7-for	GTATGGCACATCATTTACGCTTGG	905
	OST7-rev	AGCACAGACTGGTCATGGTGGTAC	
	OST8-for	GACTGTAGACTGAGGAGGTTGTTG	575
	OST8-rev	TTCTGTTACTGTTTGGCTGTTTCG	
	OST9-for	TTTTAACAAGGCAAAGGGAATGGG	616
	OST9-rev	TCAGCCTGCTTTCTTCTGTCACCC	
	OST10-for	ATGGCTGGGCGGATGAGCGGAGTA	432
	OST10- rev	CATGGGTCAGCAGGCAGAGTGGAT	
